# Systematic review of interventions for depression and anxiety in persons with inflammatory bowel disease

**DOI:** 10.1186/s13104-016-2204-2

**Published:** 2016-08-12

**Authors:** Kirsten M. Fiest, Charles N. Bernstein, John R. Walker, Lesley A. Graff, Carol A. Hitchon, Christine A. Peschken, Ryan Zarychanski, Ahmed Abou-Setta, Scott B. Patten, Jitender Sareen, James Bolton, Alexander Singer, Ruth Ann Marrie, for the CIHR Team “Defining the burden and managing the effects of psychiatric comorbidity in chronic immunoinflammatory disease”

**Affiliations:** 1 Department of Internal Medicine, Max Rady College of Medicine, Rady Faculty of Health Sciences, University of Manitoba, 820 Sherbrook Street, Winnipeg, R3A1R9 Canada; 2 Department of Clinical Health Psychology, Max Rady College of Medicine, Rady Faculty of Health Sciences, University of Manitoba, 771 Bannatyne Avenue, Winnipeg, R3E3N4 Canada; 3 George and Fay Yee Centre for Health Care Innovation, University of Manitoba, 820 Sherbrook Street, Winnipeg, R3A1R9 Canada; 4 Departments of Community Health Sciences and Psychiatry, Cumming School of Medicine, University of Calgary, 3280 Hospital Drive NW, Calgary, T2N4Z6 Canada; 5 Department of Psychiatry, Max Rady College of Medicine, Rady Faculty of Health Sciences, University of Manitoba, 771 Bannatyne Avenue, Winnipeg, R3E3N4 Canada; 6 Department of Family Medicine, Max Rady College of Medicine, Rady Faculty of Health Sciences, University of Manitoba, 770 Bannatyne Avenue, Winnipeg, R3E0W3 Canada; 7 Department of Community Health Sciences, Max Rady College of Medicine, Rady Faculty of Health Sciences, University of Manitoba, 820 Sherbrook Street, Winnipeg, R3A1R9 Canada; 8 Health Sciences Centre, 820 Sherbrook Street, Winnipeg, MB R3A 1R9 Canada

**Keywords:** Depression, Anxiety, Systematic review, Inflammatory bowel disease

## Abstract

**Background:**

Depression and anxiety are common in inflammatory bowel disease (IBD) and can affect disease outcomes, including quality of life and success of disease treatment. Successful management of psychiatric comorbidities may improve outcomes, though the effectiveness of existing treatments in IBD is unknown.

**Methods:**

We searched multiple online databases from inception until March 25, 2015, without restrictions on language, date, or location of publication. We included controlled clinical trials conducted in persons with IBD and depression or anxiety. Two independent reviewers reviewed all abstracts and full-text articles and extracted information including trial and participant characteristics. We also assessed the risk of bias.

**Results:**

Of 768 unique abstracts, we included one trial of pharmacological anxiety treatment in IBD (48 participants), which found an improvement in anxiety symptoms (p < 0.001). There was a high risk of bias in this trial. We found no controlled clinical trials on the treatment of depression in persons with IBD and depression and no controlled clinical trials reporting on psychological interventions for anxiety or depression in IBD.

**Conclusions:**

Only one trial examined an intervention for anxiety in adults with IBD and no trials studied depression in adults with IBD. The level of evidence is low because of the risk of bias and limited evidence.

**Electronic supplementary material:**

The online version of this article (doi:10.1186/s13104-016-2204-2) contains supplementary material, which is available to authorized users.

## Background

Inflammatory bowel disease (IBD) is a chronic immune-mediated inflammatory disease of the gastrointestinal tract, affecting at least 1 million Americans and 2.5 million Europeans [1]. IBD emerges in children, but the peak age of onset is in the third and fourth decades [2–4]. Hence, this disease impacts affected individuals in the prime of their lives from social and work perspectives, and is associated with considerable disability [5–7].

There is a high prevalence of depression and anxiety disorders in persons with IBD; over 25 % of individuals with IBD will experience depression in their lifetime, while anxiety may affect more than 30 % [8]. Psychiatric comorbidity is associated with adverse outcomes in IBD. Depression is associated with an increased risk of disease relapse in IBD, [9] and is a risk factor for treatment failure with infliximab [10]. By managing depression and anxiety in persons with IBD outcomes may be improved, but it is unclear if treatments for depression and anxiety that are effective in the general population would be similarly effective in persons with IBD. Commonly used pharmacological treatments for depression and anxiety may be less effective in those who use anti-inflammatory therapies, [11] or lead to potentially harmful adverse effects or exacerbations in symptoms [12].

The objective of this systematic review was to identify the existing literature on controlled clinical trials of pharmacological and psychological interventions for depression and anxiety in persons with IBD.

## Methods

We conducted this systematic review according to the approach described in the Cochrane Handbook for Systematic Reviews, [13] using an a priori published protocol [14]. We report the findings according to the preferred reporting items for systematic reviews and meta-analyses (PRISMA) criteria [15].

### Populations, interventions, comparators, settings and study designs

Trials were included if conducted in persons with IBD who were depressed and/or anxious. IBD was defined according to the criteria reported in each paper. Controlled clinical trials (i.e. randomized controlled trials (RCT), controlled before and after trials) conducted in any clinical setting were eligible. Diagnoses of depression or anxiety could be based on self-report using a screening tool or a clinical interview. We examined the methods used by individual papers to identify the study populations, including the tools used, and there were no other pre-specified criteria regarding the definition of depression or anxiety. If the entire sample was <18 years old trials were excluded to reduce heterogeneity.

### Outcome measures

Our primary research question was: “What is the efficacy of pharmacological and psychological treatments for depression or anxiety in persons with IBD”. We included the following secondary outcomes based on recommendations from primary care providers and individuals living with IBD: (1) difference in fatigue scores at post-assessment between the treatment and comparison groups; (2) difference in quality of life scores at post-assessment between the treatment and comparison groups; (3) the proportion of participants achieving ≥50 % reduction in depressive or anxiety symptoms from baseline to post-assessment between the treatment and control. In all cases post-assessment was the longest reported follow-up.

The final research question: “What is the tolerability of pharmacotherapy for depression or anxiety in IBD?”, was examined according to the dose and duration of the treatment, drop-out rates, and any reported adverse effects.

### Search strategy

We developed our search strategy (Additional file 1: Appendix) with the help of a medical librarian (MF), and experts in gastrointestinal disease (CNB) and psychiatric disorders (SBP, JW, LG, KMF, JS, JB). We searched the Cochrane Database of Systematic Reviews and the following databases: Medline, EMBASE, PsycINFO, PsycARTICLES Full Text, Cochrane Central Register of Controlled Trials, CINAHL, Web of Science, and Scopus to identify RCTs and related systematic reviews. We searched Clinicaltrials.gov and the WHO trial register to identify completed or ongoing trials. We also searched the reference lists of related systematic reviews and of the included trials to identify any further studies. There were no date or language limits placed on the searches. We searched databases from inception date to March 25, 2015. The Cochrane Highly Sensitive Search Strategy was employed in MEDLINE, and variations of this filter, or other validated filters, were used for other databases.

### Study selection process

EPPI-Reviewer [16] was employed for the two-phase title and abstract review by two independent reviewers (KMF and RAM). We reviewed titles and abstracts in the first phase to determine if they were conducted in individuals with IBD and who had depression or anxiety. In the second phase, these abstracts were determined to be controlled clinical trials or not. Following the two-phase review, the full-text articles were reviewed in detail by the same reviewers to ensure all inclusion criteria were met, and disagreements were resolved by discussion.

### Data extraction and management

We completed data extraction in duplicate using a data collection tool developed by the author team and implemented in EPPI-Reviewer. Information on study design, inclusion criteria for the study population, including demographic or disease characteristics (e.g. age, sex, disease subtype, race/ethnicity), methods for identifying psychiatric comorbidity (e.g. diagnostic interview, self-report questionnaire) including the tools used, interventions employed, items related to the risk of bias assessment (see below), and any efficacy or safety outcomes was collected.

### Risk of bias assessment and grading the evidence

Two reviewers independently evaluated the internal validity of the trials using the Cochrane Collaboration’s Risk of Bias tool [17], which evaluates risk of bias in six domains: sequence generation, allocation concealment, masking/blinding of participants, personnel, and outcome assessors, incomplete outcome data, selective outcome reporting, and other sources of bias. Each domain is rated as having either a low risk of bias, unclear risk of bias, or high risk of bias. The overall assessment is based on responses to individual domains; the overall score was rated as having a high risk of bias if one or more individual domains is assessed as having a high risk of bias. Only if all components are rated as having a low risk of bias is the overall risk rated as low. Risk of bias for all other studies was rated as unclear. Disagreements on the bias assessment were resolved by discussion. The approach described by the GRADE working group was used to determine the strength of the evidence: low, moderate, or high [18].

### Data synthesis and analysis

We used descriptive statistics, including frequencies (reported as a percent), to summarize the study findings.

## Results

### Results of the search

**Fig. 1:**
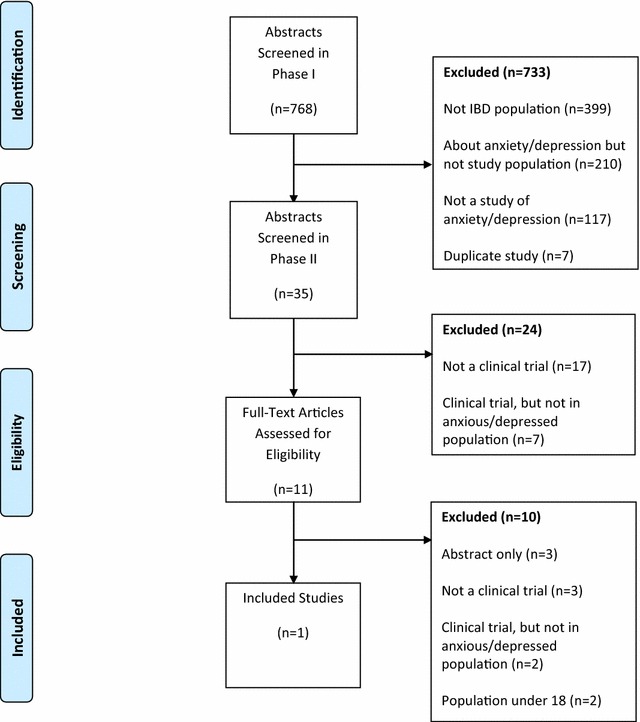
Study flow diagram

We screened 768 unique abstracts, initially excluding 733. Over 50 % (399/733) were excluded because they were not conducted in a population of persons with IBD, almost 29 % (210/733) were excluded as the study population did not have depression or anxiety, while 16 % (117/733) were excluded as they did not study depression or anxiety, and the final abstracts were excluded because they were duplicates (Fig. 1). A second abstract review was completed where a further 17 abstracts were excluded because they were not controlled clinical trials; others were excluded as they were not conducted in a population with depression or anxiety. We reviewed the full-text of 11 published manuscripts. Of these, one article met the final inclusion criteria for the review (Table 1).

**Table 1:** Included studies

Author (year) Recruitment setting	Study information	Psychiatric comorbidity information	Intervention			Outcomes		
			Intervention	Comparator	Duration	Anxiety	Outcome	Tolerability
Stokes [19] *Setting* Specialty Clinic	*Study design* Randomized controlled trial *Study population* Chronic Enteritis or Ulcerative Colitis	*Screening tool* Hamilton Anxiety Rating Scale (HAM) Moderate to severe anxious mood, and 3 anxiety-related symptoms on HAM Global rating scaleModerate anxiety	*Pharmacologic* Lorazepam 1 mg QAM 2 mg QHS (n = 24) *Provider delivering intervention* Gastroenterologist	Placebo (n = 21)	4 weeks	Hamilton Anxiety Rating Scale Lipman-Rickels 35-item Self-rating scale Global rating scale	Difference in mean scores	Discontinuation rate Rate of adverse effects

### Description of trials

A single American RCT of the treatment of anxiety in 48 persons with IBD was published in 1978 [19]. This trial assessed the effect of a pharmacological intervention (lorazepam) administered by a gastroenterologist, and was performed in a specialty clinic (Table 1). No trial of depression in IBD was identified. No controlled clinical trials of psychological interventions for anxiety or depression in IBD were found.

### Interventions and assessments

The sole trial of anxiety used the Hamilton and the Global to determine participant eligibility. The active treatment phase of this trial was 4 weeks, and participants received either lorazepam (1 mg before noon, 2 mg at bedtime) or placebo. The Hamilton Scale and Global were also used to assess changes in anxiety symptoms over time.

### Risk of bias assessment

The single included IBD trial had a high risk of bias, as there was a high risk of incomplete reporting of outcome data (Additional file 2).

### Primary outcomes

#### Depression

Depression was not reported as an outcome.

#### Anxiety

In the one trial of pharmacological anxiety treatment in IBD, anxiety scores improved between visits in the intervention group (SMD could not be calculated- treatment group mean change score: 9.2 [standard error (SE): 1.5]; placebo group mean change score: 4.0 (SE: 1.1); p < 0.01).

#### Strength of evidence

Single RCT was rated as providing moderate evidence, but we downgraded the overall strength of evidence to low because in the presence of one trial uncertainty persists regarding the true effect (Additional file 3).

### Secondary outcomes

We were unable to assess any of the secondary outcomes related to fatigue and quality of life because they were not assessed in the single included trial.

#### Tolerability

Reported adverse medication effects were numerous in both the treatment and comparison groups (Additional file 4). The most common adverse events in the treatment group were unsteadiness/incoordination (11.5 %) and dizziness, headache, sedation, dermatological symptoms, or gastrointestinal symptoms (each 7.7 %). In the comparison group dizziness, headache, sleep disturbance, nausea, and vomiting were equally common (each 4.5 %). The dropout rate in the treatment group was 7.7 % and in the comparison group 4.5 %.

## Discussion

In our systematic review of the literature, we found no trials reporting on interventions for depression in IBD, and only a single RCT reporting on an intervention for anxiety. This trial reported a statistically significant reduction in anxiety symptoms following pharmacological intervention. The trial did not report on the effects of the intervention on fatigue, pain or quality of life.

Depression and anxiety are highly prevalent in IBD [8] and can have detrimental effects on IBD disease course and quality of life. In persons with Crohn’s disease, depression or generalized anxiety disorder may be associated with an increased risk of surgery [20]. In a broader IBD population, depressive symptoms were associated with an increased number of relapses and with a shorter time to first relapse, while anxiety and reduced quality of life were associated with more frequent relapses [9]. In both ulcerative colitis and Crohn’s disease, depression has been associated with clinical disease recurrence; disease recurrence may also be associated with symptoms of anxiety in persons with Crohn’s disease [21]. In active Crohn’s disease, depression is associated with a failure to achieve remission on infliximab and earlier retreatment [10]. Regardless of disease severity, the presence of psychiatric comorbidities in IBD may contribute to poor quality of life [22].

Psychiatric comorbidities can be successfully managed in chronic inflammatory conditions, [23] and as such may be a means of improving outcomes in persons with IBD. Despite this, depression and anxiety disorders are under-recognized and undertreated in persons with IBD; one study estimated that of 43 % of persons with symptoms of depression or anxiety, only 18 % of those people were receiving psychological treatment and 21 % were taking psychotropic medications [24]. As a first step to improved management, improved detection of depression and anxiety in IBD is required.

The anxiety screening tools used (Hamilton and the Global) have not been validated for use in persons with IBD. It is imperative that depression and anxiety screening tools be validated in disease-specific settings to ensure scale performance is adequate, investigate potential confounding effects, including those of somatic symptoms, and assess the optimal scoring criteria. In other chronic diseases such as multiple sclerosis and rheumatoid arthritis, it has been suggested that the inclusion of physical symptoms of the disease (e.g. tingling, dizziness) in psychiatric screening tools may lead to “criterion contamination” [25–27]. This contamination results from an overlap in disease-specific and anxiety symptoms, which may cause people to screen positive for anxiety (i.e. an increase in false positives) when the anxiety symptoms are due to the disease itself. These screening tools may also perform differently in persons with IBD than the general population (due to criterion contamination, common pathophysiology such as inflammation [28]), and scoring cutpoints appropriate for IBD must be established.

Controlled clinical trials that did not have depression or anxiety as inclusion criteria and those with only general education approaches regarding IBD were excluded from this review. In a recent RCT, both CBT and supportive nondirective therapy significantly reduced depressive symptoms and improved global functioning, quality of life, and disease activity in 178 adolescents with depression and Crohn’s disease or ulcerative colitis [29]. In addition, the ongoing HAPPY-IBD trial assesses the impact of IBD-specific CBT on subsyndromal symptoms of depression or anxiety in adolescents with IBD [30]. These trials of adolescents may provide insight into the design and conduct of future trials in adults with IBD.

To determine the efficacy of psychosocial or pharmacological interventions for reducing clinical anxiety or depression in persons with IBD, RCTs must be appropriately designed, including targeting the appropriate population. Participants should meet diagnostic criteria for anxiety or mood disorders using validated measurement tools. Tools used to measure anxiety and depression outcomes should be shown to be valid, reliable, and sensitive to change in IBD before being employed in a trial of this population. Given the potentially broad effects of psychiatric comorbidity on outcomes in IBD, such trials should also include outcomes related to IBD disease activity, and patient-centred outcomes such as pain, fatigue, and quality of life.

This review was conducted according to established systematic review methodology using a registered protocol, searching multiple databases with limited publication restrictions. The conclusions in this paper are limited by the nature of the available literature: only one study met all inclusion criteria, and was of strength and risk of bias. The included study was published almost 40 years ago and conceptions of IBD and psychiatric disorders have changed greatly since that time. All relevant outcomes (quality of life, fatigue, disease activity, etc.) must be examined in future trials of treatments for depression or anxiety in IBD, as existing evidence suggests that they have a substantial impact on functioning.

## Conclusions

There is a dearth of evidence regarding treatment of depression or anxiety in persons with IBD. Considering the prevalence and impact these psychiatric comorbidities can have on the outcome of IBD, intervention trials in this area are desperately needed.

## Additional files

10.1186/s13104-016-2204-2 Search Strategy.
10.1186/s13104-016-2204-2 Risk of Bias Assessment.
10.1186/s13104-016-2204-2 GRADE Rating.
10.1186/s13104-016-2204-2 Medication Adverse Effects.

## Abbreviations

IBD: inflammatory bowel disease
PRISMA: preferred reporting items for systematic reviews and meta-analyses
RCT: randomized control trial
