# Supplementary material for: Systematic review of interventions for depression and anxiety in persons with inflammatory bowel disease
Source: BMC Res Notes. 2016 Aug 12;9:404. doi: 10.1186/s13104-016-2204-2 (PMC4982207; doi:10.1186/s13104-016-2204-2)
Supplement: Supplementary file 1 — 10.1186/s13104-016-2204-2 Search Strategy. [file 13104_2016_2204_MOESM1_ESM.pdf]

EMBASE

1. colonic disease/ or gastrointestinal disease/ or inflammatory bowel disease/
2. IBD.ti.ab
3. inflammatory bowel.ti.ab.
4. (ulcerative colitis or crohn$ disease$ or colitis gravis or idiopathic proctocolitis).ti.ab.
5. or/1-4 [IBD]
6. major depression/ or recurrent brief depression/ or *treatment resistant depression/
7. *dysthymia/ or *DYSPHORIA/
8. *reactive depression/ or *endogenous depression/
9. depressive.ti,ab. or depression.ti. or depressed.ti.
10. treatment resistant depress$.ab.
11. dysthym$.ti,ab.
12. dysphori$.ti,ab.
13. melancholia$.ti,ab.
14. depress$ disorder?.ti,ab.
15. major depress$.ab.
16. (recurr$ depress$ or chronic depress$).ab.
17. (anxiety adj2 depress$).ab.
18. anxiety disorder/ or "mixed anxiety and depression"/ or generalized anxiety disorder/
19. anxiety disorder?.ti,ab.
20. (chronic$ adj2 (anxious$ or anxie$)).ti,ab.
21. *acute stress disorder/
22. ((mood or mental or anxiety) adj2 (disorder? or illness?) adj9 (detect$ or diagnose? or diagnos?s or identif$ or treat$ or underdiagnos$ or under diagnos$)).ti,ab.
23. or/6-22 [Depression/Anxiety Disorders]
24. *mood disorder/
25. *panic/
26. *posttraumatic stress disorder/
27. Phobic disorders/
28. *obsessive compulsive disorder/
29. ((mood or phobic or stress) adj2 disorder?).ti,ab.
30. or/24-29 [Other Psych Disorders]
31. 5 and 23 [IBD & Depression/Anxiety]
32. (5 and 30) not 31 [IBD & Other Psych Disorders]
33. (systematic review or metanalys$ or meta-analys$ or overview or synthesis).ti.
34. "systematic review"/
35. meta analysis/
36. or/33-35 [Systematic Review Filter]
37. controlled clinical trial/ or controlled study/ or randomized controlled trial/ [EM]
38. randomi?ed.ti. or ((random$ or control) adj3 (group? or cohort? or patient? or hospital$ or department?)).ab. or (controlled adj2 (study or trial)).ti.
39. (multicenter and (study or trial)).ti.
40. (random sampl$ or random digit$ or random effect$ or random survey or random regression).ti,ab. not randomized controlled trial/ [Per BMJ Clinical Evidence Filter]
41. (exp animals/ or exp invertebrate/ or animal experiment/ or animal model/ or animal tissue/ or animal cell/ or nonhuman/) and (human/ or normal human/ or human cell/)
42. (exp animals/ or exp invertebrate/ or animal experiment/ or animal model/ or animal tissue/ or animal cell/ or nonhuman/) not 41
43. (or/37-39) not (or/40,42) [RCT Filter for EMBASE]
44. (31 or 32) and 36 [IBD Systematic reviews to export]
45. ((31 or 32) and 43) not 44 [IBD Trials to export]

Medline

1. colonic diseases/ or gastrointestinal diseases/ or inflammatory bowel diseases/
2. IBD.ti.ab.
3. inflammatory bowel.ti.ab
4. (ulcerative colitis or crohn$ disease$ or colitis gravis or idiopathic proctocolitis).ti.ab.
5. or/1-4 [IBD]
6. Depressive Disorder/ or Depressive Disorder, Major/ or depressive disorder, treatment resistant/
7. dysthymic disorder/
8. depressive.ti,ab. or depression.ti. or depressed.ti.
9. treatment resistant depress$.ab.
10. dysthym$.ti,ab.
11. dysphori$.ti,ab.
12. melancholia$.ti,ab.
13. depress$ disorder?.ti,ab.
14. major depress$.ab.
15. (recurr$ depress$ or chronic depress$).ab.
16. (anxiety adj2 depress$).ab.
17. anxiety disorders/
18. anxiety disorder?.ti,ab.
19. (chronic$ adj2 (anxious$ or anxie$)).ti,ab.
20. Stress, psychological/di, pc or (stress, psychological/ and (diagnose? or diagnos?s or underdiagnos$ or under diagnos$).ti.)
21. ((mood or mental or anxiety) adj2 (disorder? or illness?) adj9 (detect$ or diagnose? or diagnos?s or identif$ or treat$ or underdiagnos$ or under diagnos$)).ti,ab.
22. or/6-21 [Depression/Anxiety Disorders]
23. *Mental disorders/
24. mood disorders/
25. panic disorder/
26. stress disorders, traumatic/ or stress disorders, post-traumatic/
27. Phobic disorders/
28. obsessive-compulsive disorder/
29. ((mood or phobic or stress) adj2 disorder?).ti,ab.
30. or/23-29 [Other Psych Disorders]
31. 5 and 22 [IBD & Depression/Anxiety]
32. (5 and 30) not 31 [IBD & Other Psych Disorders]
33. (randomized controlled trial or controlled clinical trial).pt. or randomized.ab. or placebo.ab. or clinical trials as topic.sh. or randomly.ab. or trial.ti.
34. exp animals/ not humans.sh.
35. 33 not 34
36. (systematic review or metanalys$ or meta-analys$ or overview or synthesis).ti.
37. review.pt. and (medline or pubmed or embase or cochrane).ab.
38. (medline or systematic review).tw. or meta-analysis.pt. or intervention.ti.
39. (31 or 32) and (36 or 37 or 38) [IBD Systematic reviews to export]
40. ((31 or 32) and 35) not 39 [IBD Trials]

Psycarticles

1. IBD.ti,ab.
2. inflammatory bowel.ti,ab.
3. (ulcerative colitis or crohn$ disease$ or colitis gravis or idiopathic proctocolitis).ti.ab.
4. 1 or 2 or 3 [IBD]
5. randomi?ed.ti. or ((random$ or control) adj3 (group? or cohort? or patient? or hospital$ or department?)).ab. or (controlled adj2 (study or trial)).ti.
6. (multicenter and (study or trial)).ti.
7. ((systematic adj2 review) or overview or metaanalys$ or meta-analys$).ti.
8. 5 or 6 [Trials]
9. (4 and 8) not 7

PsycInfo-ProQuest

1. colon disorders/ or gastrointestinal disorders/ or ulcerative colitis/ or bowel disorders/
2. TI(IBD)
3. AB(IBD)
4. AB,TI(inflammatory bowel)
5. AB,TI(ulcerative colitis or crohn? disease?)
6. AB,TI(colitis gravis or idiopathic proctocolitis)
7. S1 or S2 or S3 or S4 or S5 or S6 [IBD]
8. Depressive Disorder/ or Depressive Disorder, Major/ or Dysthymic Disorder/ or Seasonal Affective Disorder/
9. Depression/ or Anxiety/ [Behavioural Symptoms/Emotions]
10. anxiety disorders/ OR exp obsessive-compulsive disorder/ OR panic disorder/ OR phobic disorders/ OR traumatic stress disorders/ OR posttraumatic stress disorders/
11. AB,TI(depression or depressive or anxiety)
12. AB(depression)
13. AB(depressive)
14. AB(anxiety)
15. S8 or S9 or S10 or S11 or S12 or S13 or S14 [Depression or Anxiety]
16. Mental health/
17. AB,TI((emotional or mental or psychological) NEAR/2 (well-being or health))
18. AB,TI((mental NEAR/2 (health or distress)) or psychological distress)
19. TI(coping or cope)
20. AB(coping NEAR/2 (behavio?r? or mechanism? or strateg* or pattern?))
21. S16 or S17 or S18 or S19 or S20 [Mental/emotional health*]
22. Mood disorders/
23. AB,TI(mood disorder?)
24. S22 or S23 [Mood Disorders]
25. Psychological stress/
26. AB,TI(stress NEAR/2 (mental or psychological or long-term or chronic or ongoing))
27. AB,TI(stressful or stressor?)
28. S25 or S25 or S27 [Stress psychological]
29. Physiological stress/
30. AB,TI(psychophysical)
31. S29 or S30 [Stress, physiological]
32. S7 and S15 [IBD & Depression]
33. (S7 and S21) not S32 [IBD & Mental Health]
34. (S7 and S24) not (S32 or S33) [IBD & Mood Disorders]
35. (S7 and S28) not (S32 or S33 or S34) [IBD & Mental Stress]
36. AB,TI("double-blind" or "random* assigned" or control)
37. (S32 or S33 or S34 or S35) and S36

SCOPUS

colon disorders/ or gastrointestinal disorders/ or ulcerative colitis/ or bowel disorders/
TITLE{inflammatory bowel}
ABS{inflammatory bowel}
TITLE ({ulcerative colitis} or {crohn? disease?})
ABS ({ulcerative colitis} or {crohn? disease?})
TITLE ({colitis gravis} or {idiopathic proctocolitis})
ABS({colitis gravis} or {idiopathic proctocolitis})
#1 or #2 or #3 or #4 or #5 or #6 or #7 [IBD]
Depressive Disorder/ or Depressive Disorder, Major/ or Dysthymic Disorder/ or Seasonal Affective Disorder/
Depression/ or Anxiety/ [Behavioural Symptoms/Emotions]
anxiety disorders/ OR obsessive-compulsive disorder/ OR panic disorder/ OR phobic disorders/ OR traumatic stress disorders/ OR posttraumatic stress disorders/
TITLE(depression or depressive or anxiety)
ABS (depression or depressive or anxiety)
#9 or #10 or #11 or #12 or #13 [Depression or Anxiety]
Mental health/
TITLE((emotional or mental or psychological) W/2 (well-being or health))
ABS ((emotional or mental or psychological) W/2 (well-being or health))
TITLE ((mental W/2 (health or distress)) or {psychological distress})
ABS ((mental W/2 (health or distress)) or {psychological distress})
TITLE(coping or cope)
ABS(coping W/2 (behavio?r? or mechanism? or strateg* or pattern?))
#15 or #16 or #17 or #18 or #19 or #20 or #21 [Mental/emotional health*]
Mood disorders/
TITLE {mood disorder?}
TITLE {mood disorder}
ABS {mood disorder}
#23 or #24 or #25 or #26 [Mood Disorders]
Psychological stress/
TITLE (stress W/2 (mental or psychological or long-term or chronic or ongoing))
ABS (stress W/2 (mental or psychological or long-term or chronic or ongoing))
TITLE (stressful or stressor?)
ABS (stressful or stressor?)
#28 or #29 or #30 or #31 or #32 [Stress psychological]
#8 and #14 [IBD & Depression]
(#8 and #22) and not #34 [IBD & Mental Health]
(#8 and #26) and not (#34 or #35) [IBD & Mood Disorders]
(#8 and #33) and not (#34 or #35 or #36) [IBD & Mental Stress]
TITLE ("double-blind" or "random* assigned" or control)
ABS ("double-blind" or "random* assigned" or control)
(#34 or #35 or #36 or #37) and (#38 or #39)
#40 and SUBJAREA(mult or neur or phar or medi or nurs or heal or psyc)

TS= (colon disorders or gastrointestinal disorders or ulcerative colitis or bowel disorders)

TI = (inflammatory bowel)

TI= (ulcerative colitis)

TI=(crohn* disease*)

TS=(crohn* disease*)

TI=(colitis gravis)

TS=(colitis gravis)

TI=(idiopathic proctocolitis)

TS=(idiopathic proctocolitis)

TS=(depress* OR anxiety)

TI=(depress* OR anxiety)

TS=(major depress* disorder)

TI=(major depress* disorder)

TS=(dysthymi*)

TI=(dysthymi*)

TS=(major depress*)

TI=(major depress*)

TS=(seasonal affective disorder)

TI=(seasonal affective disorder)

TS=(obsessive compulsive disorder)

TI=(obsessive compulsive disorder)

TS=(panic disorder)

TI=(panic disorder)

TS=(phobi*)

TI=(phobi*)

TI=(posttraumatic stress)

TS=(posttraumatic stress)

TS=(mood disorder*)

TI=(mood disorder*)
