# Supplementary material for: Systematic review of interventions for depression and anxiety in persons with inflammatory bowel disease
Source: BMC Res Notes. 2016 Aug 12;9:404. doi: 10.1186/s13104-016-2204-2 (PMC4982207; doi:10.1186/s13104-016-2204-2)
Supplement: Supplementary file 2 — 10.1186/s13104-016-2204-2 Risk of Bias Assessment. [file 13104_2016_2204_MOESM2_ESM.docx]

Risk of Bias Assessment

| **Study** | **Sequence Generation** | **Allocation Concealment** | **Blinding** | **Incomplete Outcome Reporting** | **Selective Outcome Reporting** | **Other Sources of Bias** | **Overall Risk of Bias** |
| --- | --- | --- | --- | --- | --- | --- | --- |
| Stokes (1978) | Unclear | Unclear | Low | High | Unclear | Unclear | High |
