# Supplementary material for: Systematic review of interventions for depression and anxiety in persons with inflammatory bowel disease
Source: BMC Res Notes. 2016 Aug 12;9:404. doi: 10.1186/s13104-016-2204-2 (PMC4982207; doi:10.1186/s13104-016-2204-2)
Supplement: Supplementary file 3 — 10.1186/s13104-016-2204-2 GRADE Rating. [file 13104_2016_2204_MOESM3_ESM.docx]

GRADE Rating

| **Study (year)** | **Study Design** | **Risk of Bias** | **Inconsistency** | **Indirectness** | **Imprecision** | **Other Considerations** | **Quality** |
| --- | --- | --- | --- | --- | --- | --- | --- |
| Stokes 1978 | RCT | Serious | Not Serious | Not Serious | Not Serious | None | ⨁⨁⨁◯ MODERATE |
