# Supplementary material for: Systematic review of interventions for depression and anxiety in persons with inflammatory bowel disease
Source: BMC Res Notes. 2016 Aug 12;9:404. doi: 10.1186/s13104-016-2204-2 (PMC4982207; doi:10.1186/s13104-016-2204-2)
Supplement: Supplementary file 4 — 10.1186/s13104-016-2204-2 Medication Adverse Effects. [file 13104_2016_2204_MOESM4_ESM.docx]

Medication Adverse Effects

| **Study** | **Drop Out (%)** | | **Adverse Event** | **Rate of Adverse Effects (%)** | |
| --- | --- | --- | --- | --- | --- |
| Stokes (1978) | *Lorazepam* | *Placebo* |  | *Lorazepam* | *Placebo* |
|  | 7.7 | 4.5 | Unsteadiness and incoordination | 11.5 | 0.0 |
|  |  | | Dizziness | 7.7 | 4.5 |
|  |  |  | Disorientation or mental impairment | 3.8 | 0.0 |
|  |  |  | Headache | 7.7 | 4.5 |
|  |  |  | Sleep disturbance | 0.0 | 4.5 |
|  |  |  | Sedation | 7.7 | 0.0 |
|  |  |  | Dermatological symptoms | 7.7 | 0.0 |
|  |  |  | Nausea | 3.8 | 4.5 |
|  |  |  | Vomiting | 3.8 | 4.5 |
|  |  |  | Gastrointestinal symptoms | 7.7 | 0.0 |
